# Progesterone attenuates neurological deficits and exerts a protective effect on damaged axons via the PI3K/AKT/mTOR-dependent pathway in a mouse model of intracerebral hemorrhage

**DOI:** 10.18632/aging.203954

**Published:** 2022-03-19

**Authors:** Chang Liu, Weina Gao, Long Zhao, Yi Cao

**Affiliations:** 1 Department of Neurosurgery, West China Medical School, West China Hospital, Sichuan University, Chengdu 610041, Sichuan Province, P.R. China; 2 Department of Intensive Care Unit, The Affiliated Chengdu 363 Hospital of Southwest Medical University, Chengdu 610041, Sichuan Province, P.R. China; 3 Department of Neurosurgery, Affiliated Hospital of North Sichuan Medical College, Nanchong 637002, Sichuan Province, P.R. China; 4 Department of Neurosurgery, Chengdu Second People’s Hospital, Chengdu 610021, Sichuan Province, P.R. China

**Keywords:** intracerebral hemorrhage, axonal regeneration, progesterone, neurological function

## Abstract

Intracerebral hemorrhage (ICH) is a devastating event with high disability and fatality rates. However, there is a lack of effective treatments for this condition. We aimed to investigate the neuroprotective and axonal regenerative effects of progesterone after ICH. For this purpose, an ICH model was established in adult mice by injecting type VII collagenase into the striatum; the mice were then treated with progesterone (8 mg/kg). Hematoma absorption, neurological scores, and brain water content were evaluated on days one, three, and seven after the ICH. The effect of progesterone on inflammation and axonal regeneration was examined on day three after the ICH using western blotting, immunohistochemistry, immunofluorescence, as well as hematoxylin-eosin, Nissl, and Luxol fast blue staining. In addition, we combined progesterone with the phosphoinositide 3-kinase/serine/threonine-specific protein kinase (PI3K/AKT) inhibitor, LY294002, to explore its potential neuroprotective mechanisms. Administration of progesterone attenuated the neurological deficits and expression of inflammatory cytokines and promoted axonal regeneration after ICH, this effect was blocked by LY294002. Collectively, these results suggest that progesterone could reduce axonal damage and produced partial neuroprotective effects after ICH through the PI3K/AKT/mTOR pathway, providing a new therapeutic target and basis for the treatment of ICH.

## INTRODUCTION

Intracerebral hemorrhage (ICH) is a common and severe cerebrovascular condition associated with a considerable risk of disability and a high mortality rate within one month of the event. It accounts for approximately 15–20% of all strokes, affecting more than 2,000,000 people per year worldwide [1–3]. With the aging of the population, the incidence of ICH is increasing [4]. Currently, there is no effective surgical or pharmacological treatment for ICH. In addition, patients often experience severe neurological dysfunction after surgery, which affects their quality of life. Therefore, it is particularly urgent to find reliable and effective methods to treat ICH.

Hematoma enlargement and its compressive effect are the main causes of primary brain injury after ICH, causing half of all deaths [5]. Secondary brain injuries caused by parenchymal blood, inflammatory response, and oxidative stress are also key factors affecting the prognosis [6]. Previous studies have shown that in ICH models, a series of pathophysiological processes, such as oxidative stress, neuroinflammation, and neuroexcitatory toxicity, can lead to the death of perihematomal neurons, axonal damage, and demyelination [7]. ICH weakens the regenerative ability of the white matter axons, thereby impairing neuronal function [8]. Therefore, improving axonal repair and regeneration is the key to reversing neurological dysfunction after ICH.

Progesterone is a neurosteroid hormone that has protective effects on the function and vitality of neural cells [9]; therefore, it is now considered a promising candidate for the treatment of brain injury because it reduces inflammation, oxidative stress, and apoptosis and promotes DNA repair [10–12]. Previous studies have shown that progesterone inhibits acute nervous system injuries, such as traumatic brain injury (TBI), hypoxic-ischemic brain damage, and spinal cord injury [13–15]. Moreover, there is evidence that progesterone can improve the long-term neurological prognosis of ICH in middle-aged mice by mitigating inflammatory responses and decreasing glial scar thickness and myelin loss [16]. In addition, increasing evidence has shown that progesterone exerts protective effects in some animal models of axonal pathologies, such as autoimmune encephalomyelitis and subarachnoid hemorrhage [17, 18].

The phosphoinositide 3-kinase/serine/threonine-specific protein kinase (PI3K/AKT) pathway plays a key role in axonal regeneration and is an important protective signaling pathway in stroke [19, 20]. The mechanistic target of rapamycin (mTOR) is an important downstream target of the PI3K/AKT pathway; activating mTOR can induce protein synthesis in injured neurons, thus promoting extensive axonal regeneration [21]. Progesterone reduces inflammation and apoptosis during ischemic brain injury by activating the PI3K/AKT pathway [22]. Therefore, we speculated that progesterone might exert protective effects on damaged axons and attenuate neurological deficits through the PI3K/AKT/mTOR pathway after ICH. Previous studies have also shown that ICH induced severe blood-brain barrier disruption on day three [23]. Moreover, it has been confirmed that the markers of axonal regeneration, such as the growth-associated protein 43 (GAP43) and neurofilament 200 (NF200), changed three days after ICH, and intervention at this time point can promote axonal regeneration and improve neurological behavior [50]. Therefore, in this study, we investigated the possibly related changes at the same time point.

## RESULTS

### Progesterone reduces neurological deficits, brain water content, and pathological injury in the perihematomal region after ICH

**Figure 1:**
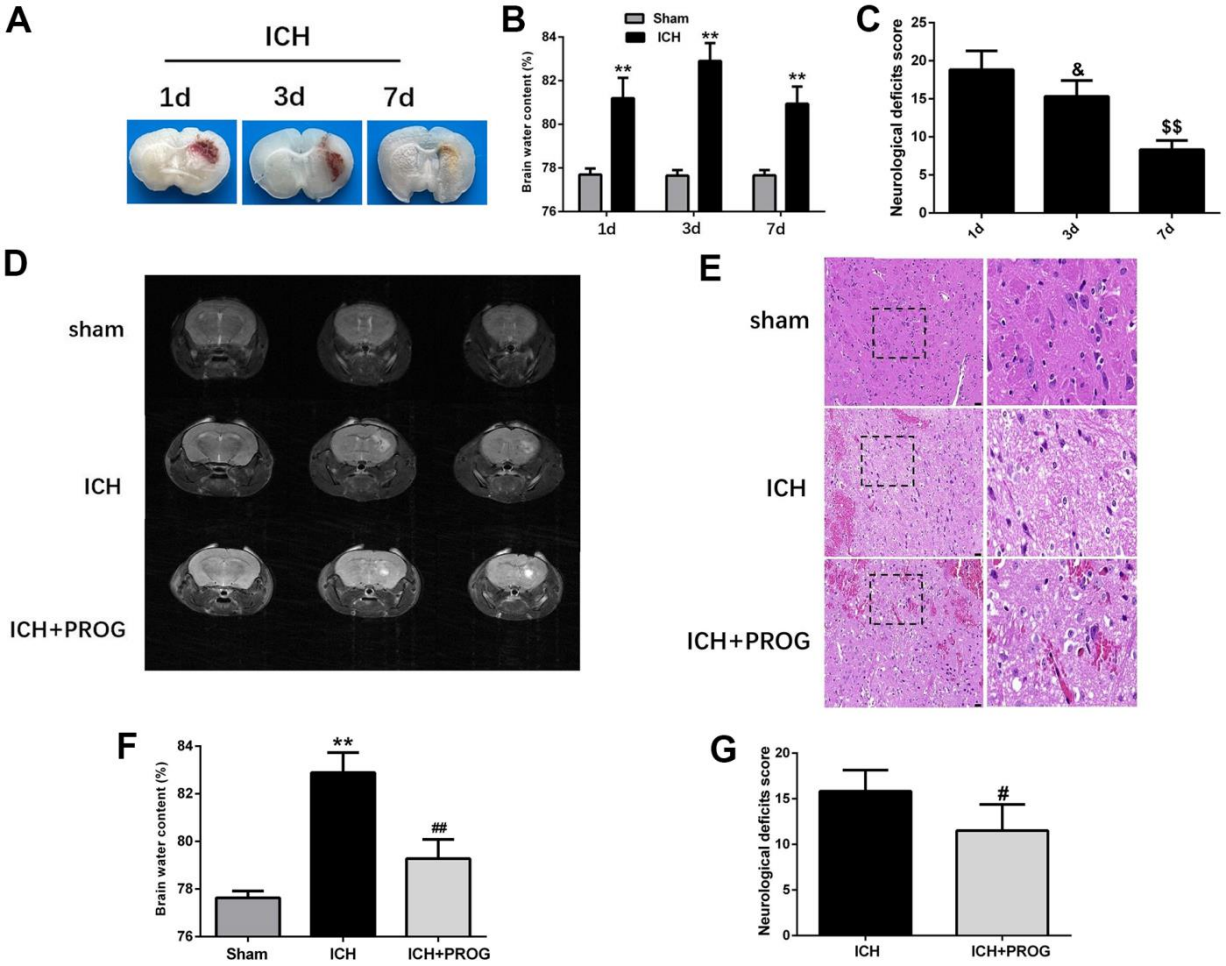
**Time-dependence trend of hematoma absorption, neurological deficit, brain water content and the neuroprotective effect of progesterone.** (**A**) Representative images of the brain tissues slices at 1, 3 and 7 days after ICH. (**B**) Brain water content at 1, 3, and 7 days after ICH. (**C**) Neurological deficit score at 1, 3, and 7 days after ICH. (**D**) Representative image of 7.0T MRI in each group. (**E**) The pathological injury was evaluated by hematoxylin-eosin (HE) staining. (**F**, **G**) On day 3 after ICH, progesterone treatment reduced brain water content and improved neurological deficit. n = 6 animals per group. Data are expressed as the mean ± SEM; **P < 0.01 vs. sham; ^#^P < 0.05 vs. ICH group; ^##^P < 0.01 vs. ICH group. ^&^P<0.05 vs. ICH 1d group; ^$$^P<0.01 vs. ICH 3d group. ICH: intracerebral hemorrhage.

We assessed neurological deficits and brain water content one, three, and seven days after ICH induction. Similar to the previous study, the brain water content was highest on the third day after ICH, and the neurological deficits improved gradually with time (Figure 1A–1C and Supplementary Table 1). Using 7.0 T MRI, we also found that progesterone could reduce the hematoma volume three days after ICH induction, compared with the control ICH group (Figure 1D). As shown in Figure 1E, intracellular vacuoles, neuron pyknosis, and partial neurocyte necrosis were found in the perihematomal region in the ICH group, while the ICH + progesterone group had a lower state of damage (Figure 1E). In addition, compared with the ICH group, the mice with progesterone treatment presented reduced brain water content (P < 0.01; Figure 1F) and neurological impairment (P < 0.05; Figure 1G and Supplementary Table 2) after ICH.

### Progesterone alleviates ICH-induced neuronal death and cell apoptosis

**Figure 2:**
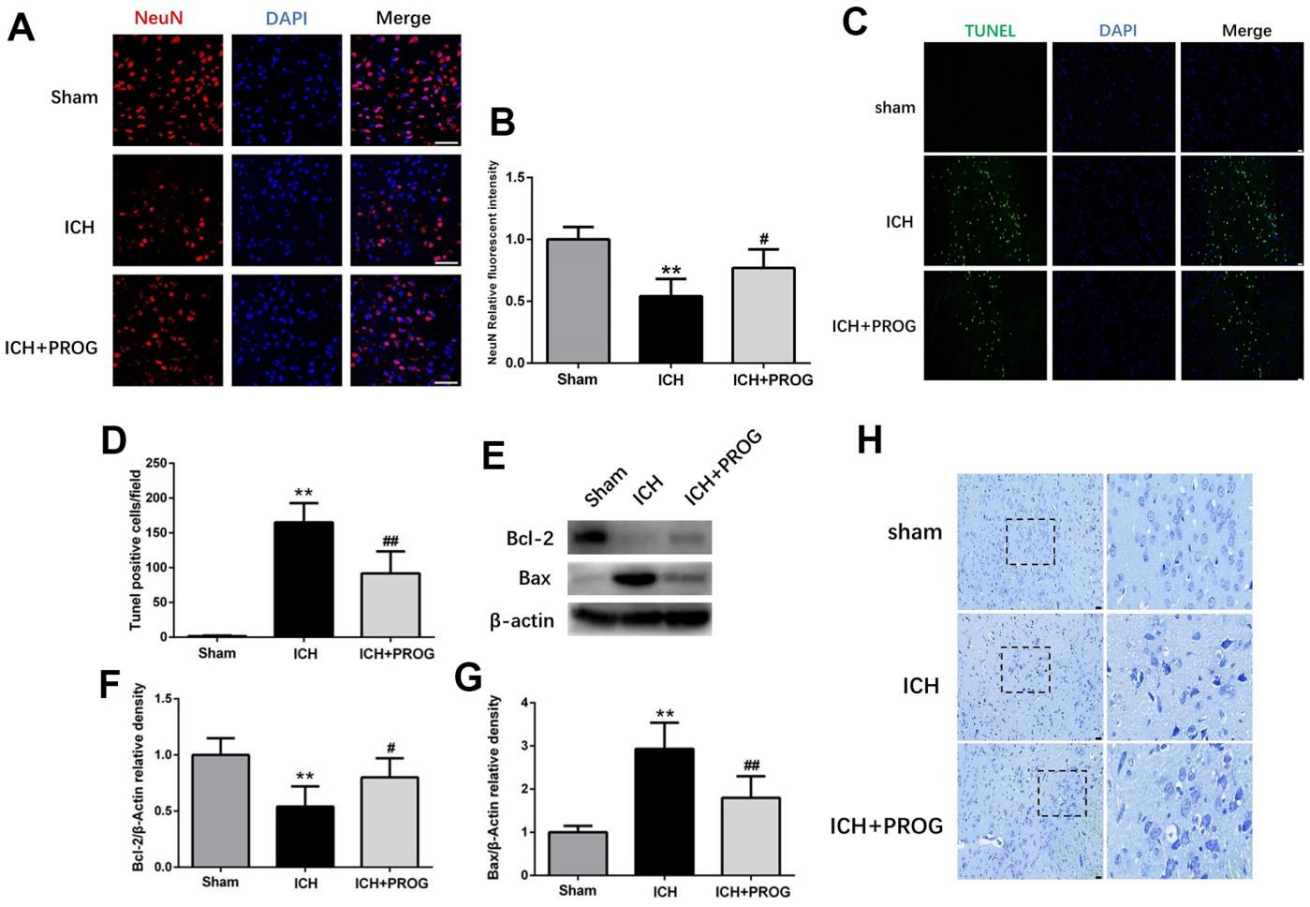
**Effect of progesterone on neuron death and cell apoptosis.** (**A**) Representative immunofluorescence staining images of NeuN (red) in perihematomal region. Nuclei were counterstained with DAPI (blue). Bar=50μm. (**B**) Quantitative analyses of NeuN relative fluorescent intensity in perihematomal region in each group. (**C**) Representative micrographs of TUNEL staining in perihematomal region in each group. Bar=50μm. (**D**) Quantitative analysis shows that TUNEL positive cells in perihematomal region. (**E**) Representative Western bands showing the protein expression of Bcl-2 and Bax in perihematomal region. (**F**, **G**) Quantitative analysis of Western blots shows that the expression of Bcl-2 and Bax changes in each group. (**H**) Representative Nissl-stained images in perihematomal region in each group. Bar=50μm. n = 6 animals per group. Data are expressed as the mean ± SEM; **P < 0.01 vs. sham; ^#^P < 0.05 vs. ICH group; ^##^P < 0.01 vs. ICH group. PROG: progesterone.

We investigated whether progesterone reduces neuronal death and apoptosis after ICH. Immunofluorescence analysis showed that neuronal nuclear protein (NeuN)-positive cells decreased after ICH and increased after progesterone treatment (P < 0.05; Figure 2A, 2B). Furthermore, the number of terminal deoxynucleotidyl transferase dUTP nick end labeling (TUNEL)-positive cells increased in the ICH group and decreased with progesterone treatment (P < 0.05; Figure 2C, 2D). In addition, progesterone treatment decreased the expression levels of BCL2 Associated X (Bax) and increased B-cell lymphoma 2 (Bcl-2) apoptosis regulator (P < 0.05; Figure 2E–2G). The results of Nissl staining showed that ICH potentially reduced the number of surviving neurons compared to the sham group, and progesterone treatment alleviated the neuronal injury (Figure 2H). These results suggest that progesterone can alleviate neuronal death and apoptosis after ICH.

### Progesterone reduces neutrophil infiltration, microglia and astrocyte activation, and inflammatory cytokines

**Figure 3:**
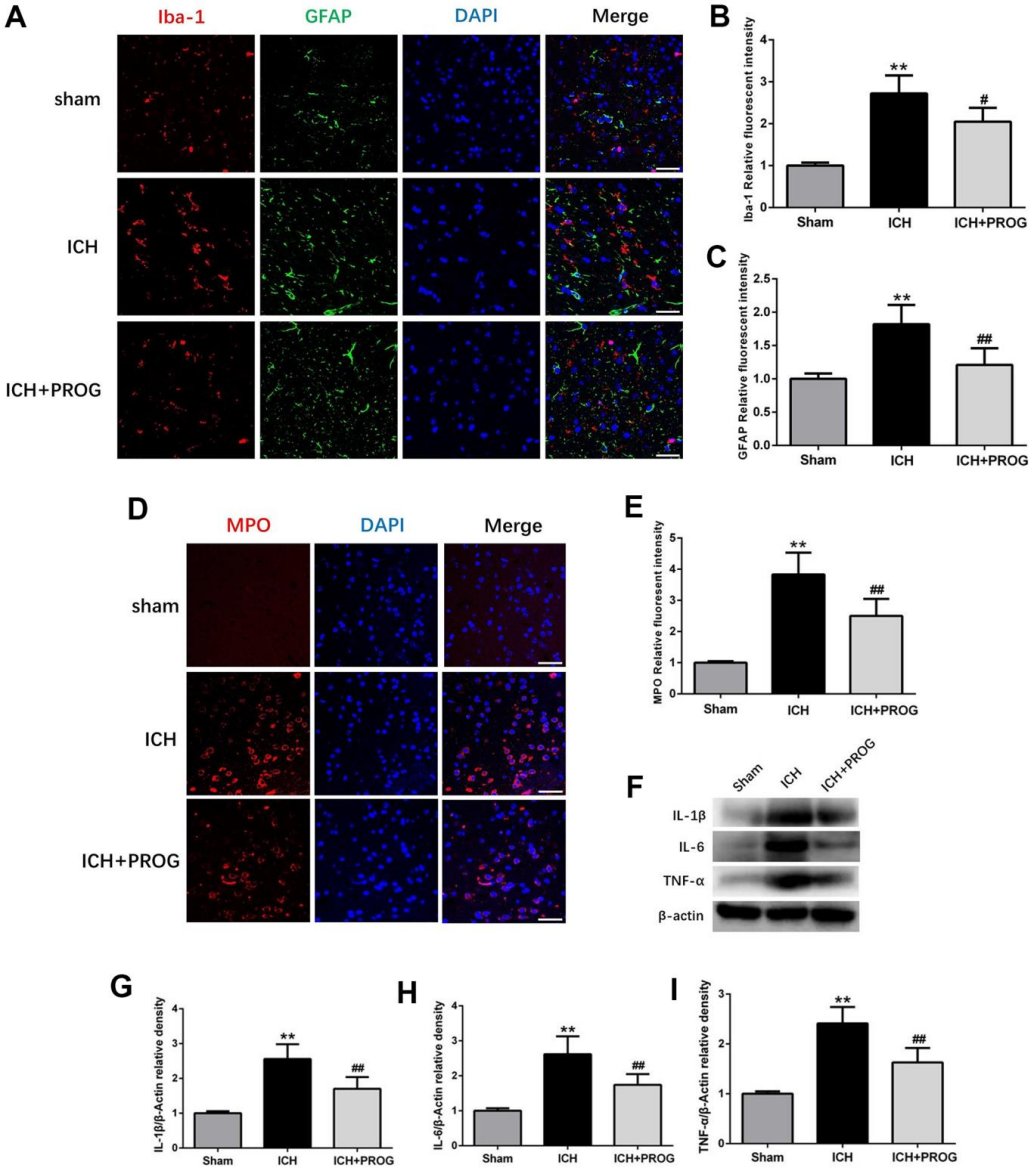
**Effect of progesterone on the activation of microglial and astrocyte, neutrophil infiltration, neuroinflammation.** (**A**) Representative immunofluorescence staining images of Iba-1(red) and GFAP (green) in perihematomal region. Nuclei were counterstained with DAPI (blue). Bar=50μm. (**B**, **C**) Quantitative analyses of Iba-1 and GFAP relative fluorescent intensity in perihematomal region in each group. (**D**) Representative immunofluorescence staining images of MPO (red) in perihematomal region. Nuclei were counterstained with DAPI (blue). Bar=50μm. (**E**) Quantitative analyses of MPO relative fluorescent intensity in perihematomal region in each group. (**F**) Representative Western bands showing the protein expression of IL-1β, IL-6, TNF-α in perihematomal region. (**G**–**I**) Quantitative analysis of Western blots shows that the expression of IL-1β, IL-6, TNF-α changes in each group. n = 6 animals per group. Data are expressed as the mean ± SEM; **P < 0.01 vs. sham; ^#^P < 0.05 vs. ICH group; ^##^P < 0.01 vs. ICH group. GFAP: glial fibrillary acidic protein; Iba-1: ionized calcium binding adapter molecule 1; MPO: myeloperoxidase.

The fluorescence intensity of the glial fibrillary acidic protein (GFAP) and ionized calcium-binding adaptor molecule 1 (Iba1) after ICH was higher than in the sham group; this effect was reversed with progesterone treatment (P < 0.05; Figure 3A–3C). Similar to previous studies, we found that myeloperoxidase (MPO) increased in the ICH group compared with the sham group and was reduced by progesterone treatment (P < 0.05; Figure 3D, 3E). In addition, we measured the expression levels of inflammatory cytokines, including interleukin (IL)-1β, IL-6, and tumor necrosis factor-alpha (TNF-α) after ICH and investigated whether progesterone intervention affected inflammation. We found that compared with the sham group, the expression of IL-1β, IL-6, and TNF-α in the ICH group was higher. However, progesterone treatment reduced the expression of inflammatory cytokines compared with the ICH group (P < 0.01; Figure 3F–3I).

### Progesterone reduces myelin loss and promotes axonal regeneration

**Figure 4:**
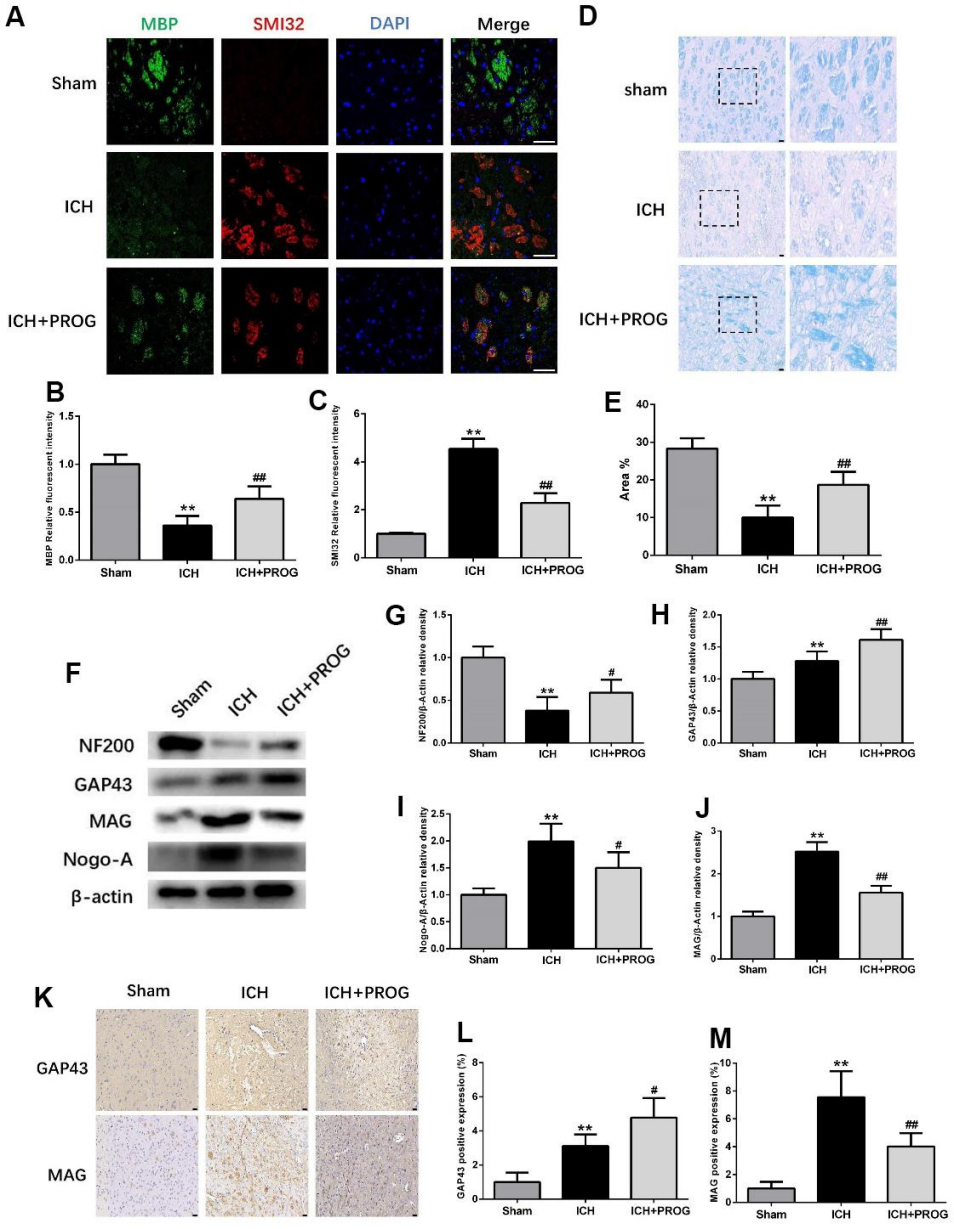
**Effect of progesterone on myelin loss and axonal pathology.** (**A**) Representative immunofluorescence staining images of MBP (green) and SMI32(red)in perihematomal region. Nuclei were counterstained with DAPI (blue). Bar=50μm. (**B**, **C**) Quantitative analyses of MBP and SMI32 relative fluorescent intensity in perihematomal region in each group. (**D**) Representative images of Luxol fast blue staining. Bar=50μm. (**E**) Quantitative analyses of positive stained myelin ratio in each group. (**F**) Representative Western bands showing the protein expression of NF200, GAP43, Nogo-A and MAG in perihematomal region. (**G**–**J**) Quantitative analysis of Western blots shows that the expression of NF200, GAP43, Nogo-A and MAG changes in each group. (**K**) Representative images of immunohistochemistry staining of GAP43 and MAG in perihematomal region. Bar=50μm. (**L**, **M**) Quantitative analyses of GAP43 and MAG positive expression in perihematomal region in each group. n = 6 animals per group. Data are expressed as the mean ± SEM; **P < 0.01 vs. sham; ^#^P < 0.05 vs. ICH group; ^##^P < 0.01 vs. ICH group. MBP: myelin basic protein; SMI32: SMI32: Stemberger Monoclonal Incorporated Antibody 32; GAP43: Growth associated protein-43; NF200: Neurofilament; Nogo-A: Neurite outgrowth inhibitor-A; MAG: Myelin Associated Glycoprotein.

Immunofluorescence staining for the myelin basic protein (MBP) and anti-neurofilament-h non-phosphorylated mouse antibody (SMI32) was performed to elucidate further the effects of progesterone on myelin loss and axonal pathology. Compared with the sham group, the MBP fluorescence intensity decreased and SMI32 increased in the ICH group, and these effects were reversed by progesterone treatment (P < 0.05; Figure 4A–4C). Luxol fast blue (LFB) was used to label normal myelin to examine the loss of this substance on day three after ICH. The results showed that progesterone treatment reduced the loss of myelin compared with the ICH group (P < 0.05; Figure 4D, 4E). Next, we examined the expression of the axonal growth factors GAP43, NF200, and the myelin inhibitors myelin-associated glycoprotein (MAG) and neurite outgrowth inhibitor (Nogo)-A. The results showed that the expression of NF200 decreased compared with the sham group, while GAP43, Nogo-A, and MAG increased in the ICH group; progesterone treatment reversed these effects (P < 0.05; Figure 4F–4J). Immunohistochemistry results further confirmed that progesterone could increase the expression of GAP43 and decrease MAG (P < 0.05; Figure 4K–4M) after ICH.

### Progesterone inhibits neuroinflammation and promotes axonal regeneration through the PI3K/AKT/mTOR pathway

**Figure 5:**
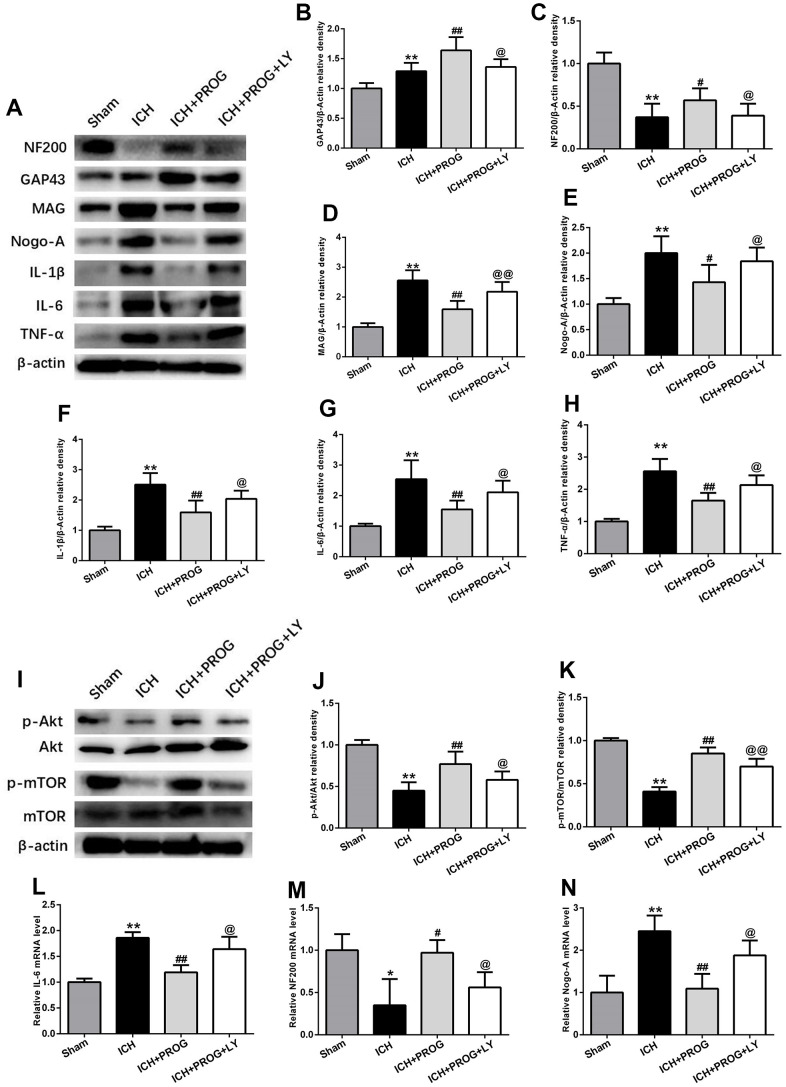
**The mechanism of the effects of progesterone on anti-inflammation and promoting axonal regeneration.** (**A**) Representative Western bands showing the protein expression of NF200, GAP43, Nogo-A, MAG IL-1β, IL-6 and TNF-α in perihematomal region.(**B**–**H**) Quantitative analysis of Western blots shows that the expression of NF200, GAP43, Nogo-A, MAG IL-1β, IL-6 and TNF-α changes in each group. (**I**) Representative Western bands showing the protein expression of p-Akt, total-Akt, p-mTOR and total-mTOR in perihematomal region. n = 6 animals per group. (**J**, **K**) Quantitative analysis of Western blots shows that the expression of p-Akt/total-Akt, p-mTOR/total-mTOR changes in each group. (**L**–**N**) mRNA expression levels of inflammatory and axon-related markers. n = 3 animals per group. Data are expressed as the mean ± SEM; *P < 0.05 vs. sham; **P < 0.01 vs. sham; ^#^P < 0.05 vs. ICH group; ^##^P < 0.01 vs. ICH group; @ P < 0.05 vs. ICH+ progesterone group; @@ P < 0.01 vs. ICH+ progesterone group. LY:LY294002.

A group of mice was additionally treated with the PI3K inhibitor LY294002 to elucidate the mechanisms of the neuroprotective effects of progesterone on inflammation and the promotion of axonal regeneration. This molecule downregulated NF200 and GAP43 and upregulated Nogo-A, MAG, IL-1β, IL-6, and TNF-α, compared to the that observed with progesterone treatment (P < 0.05; Figure 5A–5H). Similar to the results of previous studies, the PI3K/AKT/mTOR signaling pathway proteins phosphorylated AKT (p-AKT) and phosphorylated mTOR (p-mTOR) decreased after ICH. At the same time, we found that p-AKT and p-mTOR expression increased after treatment with progesterone compared with the ICH group, and this effect was blocked by LY294002 administration (P < 0.05; Figure 5I–5K). In addition, markers of inflammation and axon regeneration were detected by reverse transcription-polymerase chain reaction (RT-PCR), and similar results were obtained as before (P < 0.05; Figure 5L–5N).

## DISCUSSION

A large number of studies investigated the mechanisms of hemorrhagic brain injury. However, the pathogenesis of these injures after ICH remains unclear. Previous studies confirmed that axonal regeneration and repair played a crucial role in recovering neurological function after ICH [24, 25]. Although central axons have no regenerative capacity in adults, mechanical damage can stimulate the formation of new structural connections [26]. Studies have shown that reconstructing damaged axons and dendrites after brain injury can partially restore neurological function [27]. Therefore, an effective therapeutic strategy for ICH that restores axonal structure and function is needed. The present study revealed three major findings: (1) Progesterone could produce some neuroprotective effects including promote hematoma absorption, reduce neuronal death, cell apoptosis, inflammatory response, and activation of microglia, astrocytes, and neutrophils after ICH; (2) Treatment with progesterone reduced myelin loss and promoted axonal regeneration after ICH; (3) The mechanisms underlying the neuroprotective effect of progesterone, such as anti-inflammation and promotion of axonal regeneration after ICH, may involve the PI3K/AKT/mTOR pathway.

Progesterone reportedly acts as a neuroprotective agent via anti-oxidative, anti-inflammatory, and anti-apoptotic mechanisms [28, 29]. For example, progesterone treatment downregulates aquaporin-4 (AQP-4) in the lateral ventricles and surrounding tissues in the injured brain and significantly reduces the degree of cerebral edema after subarachnoid hemorrhage [30]. In addition, progesterone reduces the secondary degeneration in motor neurons of the spinal cord by upregulating the brain-derived neurotrophic factor [31]. Moreover, in a chronic hypoxia neonatal rat model, progesterone prevents white matter injury, improves brain maturation, and causes a switch from the state of classical activation (M1) to alternative activation/acquired deactivation (M2) in microglia [32]. At the same time, progesterone has a clear neuroprotective effect on some age-related diseases such as Alzheimer's disease, its regulation of NLRP3 inflammasome activation may be a potential therapeutic target for inhibiting astrocytic neuroinflammation in Alzheimer's disease [33]. In addition, animal experiments have confirmed that progesterone has a significant neuroprotective effect on aged male and female cerebral ischemia rats, which indicates that progesterone has great research value on aged-related nervous system diseases [34]. In recent years, progesterone was reported to have a neuroprotective effect also in ICH. Hematoma is a common neurological condition that causes a series of biological events, such as neuroinflammation, oxidative stress, glial formation, and microglial activation, which eventually lead to neuronal damage and death [35–37]. We focused on the time point of three days after ICH to verify whether progesterone alleviated these negative effects, and our findings and conclusions are consistent with previous experimental studies [16]. We found that progesterone could improve neurobehavioral deficits and reduce brain edema, neuronal death, neuroinflammation, neutrophil infiltration, microglia and astrocyte activation, and cell apoptosis. These results further confirmed the neuroprotective effects of progesterone on ICH.

However, whether the neuroprotective effect of progesterone on ICH is related to axonal regeneration and the possible mechanisms producing this effect remain unknown. The ICH induction model in rodents used in this study to simulate clinical ICH can result in white matter damage, including demyelination and axonal injury [38]. The progress of axonal injury includes a series of complex changes, including calcium influx, destruction of the neurofilament cytoskeleton, delayed rupture of the axonal membrane, and eventually irreversible axonal disconnection [39]. Some studies examined the protection from axonal injury and the improvement of neurological function, and mounting evidence suggests the presence of an early window of opportunity for axon-targeted treatment [40]. Simvastatin has been reported to reduce axonal damage and promote neurite growth in primary cortical neurons after experimental TBI [41]. A previous study showed that an adeno-associated viral vector expressing a dominant-negative form of Unc-51-like autophagy activating kinase 1 (ULK1) affects axonal regeneration in the central nervous system. This molecule may be associated with enhanced extracellular signal-regulated protein kinase 1 (ERK1) activation and reduced expression of Ras-homolog family member A (RhoA) and Rho-associated protein kinase 2 (ROCK2) [42]. Another study demonstrated that combining constitutively active Ras-homolog expressed in the brain (Rheb) and chondroitinase could promote axonal regeneration after cervical spinal cord injury [43]. Numerous studies revealed that progesterone exerts a beneficial influence on myelin repair and axonal regeneration [44–46]. However, there are no reports on whether progesterone can promote axonal regeneration after ICH. In this study, using LFB staining and immunofluorescent labeling of MBP and SMI32, we found that progesterone could reduce myelin loss three days after ICH. Moreover, our data showed that progesterone could increase the expression of GAP43 and NF200 and decrease the expression of Nogo-A and MAG after ICH. These results suggest that progesterone promotes axonal regeneration after ICH.

PI3K/AKT is a key signaling pathway involved in cell growth, metabolism, inflammation, cell survival, and other signaling events [47]. mTOR, a downstream target of the PI3K/AKT pathway, plays a vital role in axon regeneration [48]. A previous study showed that catalpol switched the intrinsic neuronal activity to promote axonal growth via the PI3K/AKT/mTOR signaling pathway after a stroke [49]. Another study confirmed that elevated microRNA 29a precursor (miR-29a) could promote axonal outgrowth and neurological recovery after ICH by activating the PI3K/AKT/mTOR pathway [50]. Additionally, leukocyte immunoglobulin-like receptor B1 (PirB) inhibits axonal outgrowth and is involved in the inhibition of the PI3K/AKT/mTOR pathway [51]. This evidence suggests that the PI3K/AKT/mTOR pathway plays a crucial role in regulating axonal regeneration in mammals. Progesterone reportedly exerts neuroprotective effects by activating the PI3K/AKT pathway [52]. We used the PI3K/AKT pathway inhibitor LY294002 to verify the underlying mechanism of this effect and confirm whether progesterone can promote axonal growth by activating the PI3K/AKT pathway and increasing mTOR after intracerebral hemorrhage. LY294002 is a selective inhibitor of PI3K activity; several studies showed that the role of LY294002 is related to the PI3K/AKT/mTOR signaling pathway [53, 54]. Studies have reported that in some nervous system diseases, such as cerebral ischemia and cortical and spinal cord injuries, specific interventional measures to promote axon regeneration can be invalidated using LY294002, which inhibits the PI3K/AKT pathway [55–57]. Therefore, we aimed verify whether LY294002 can also block the axonal regeneration induced by progesterone after ICH; this effect would confirm that activation of the PI3K/AKT/mTOR pathway plays an important role in progesterone-induced axonal regeneration. In this study, we found that mice treated with LY294002 showed decreased expression of GAP43, NF200, and the PI3K/AKT pathway proteins, p-AKT and p-mTOR. In contrast, the expression of Nogo-A, MAG, IL-1β, IL-6, and TNF-α increased compared to that in the progesterone-treated mice. These data suggest that progesterone inhibits neuroinflammation and promotes axonal regeneration by activating the PI3K/AKT/mTOR pathway after ICH.

The present study has some limitations. First, the dose of progesterone administered was identical to that used in our previous study, and we did not study the possible effects of different doses of progesterone on neurobehavioral changes and axonal regeneration after ICH. Second, we only observed the effect of progesterone on axonal regeneration three days after ICH; the neuroprotective effect of progesterone and the changes in axonal-related markers at longer time points were not examined. In addition, as in previous studies, we noticed that progesterone could reduce microglial activation. Currently, the polarization of microglia can be divided into two types: M1-type (pro-inflammatory) and M2-type (anti-inflammatory). However, we have not studied the polarization characteristics of microglia after ICH or the effects of progesterone on microglia polarization. Lastly, we did not focus on whether progesterone and LY294002 had effects, side effects or beneficial effects on non-ICH areas. Previous studies have shown that progesterone has a significant neuroprotective effect in rodent nervous system pathology models, but has little effect on the neurological function in normal mice. Espinosa-Garcia et al. showed that some indicators of neurological function changes, such as the survival of neurons, activation and polarization of microglia, and some neuroinflammatory indicators, including IL-1β, TNF-α, and NFκB, showed no significant difference between the sham group and sham + progesterone group [22, 58]. In recent years, researchers compared the locomotor activity between the sham + vehicle (sesame oil) group and the sham + progesterone group, and found no obvious difference in their locomotor activity. The team further evaluated the effects of progesterone on normal animals through familiarization and discrimination trials, and the results showed that in the familiarization trial, there was no significant difference between the sham + vehicle and sham + progesterone groups. In the discrimination trial, the novel object (%) and familiar object (%) in the sham + progesterone group increased compared with the sham + vehicle group [59]. In addition, LY294002, as a commonly used inhibitor of the PI3K/Akt pathway, has been used in ICH with relatively mature application, and it is generally believed that it will not cause side effects or benefits in non-ICH area [60, 61]. Overall, the current study showed that progesterone and LY294002 had no significant effect on the neurological function in normal animals. In summary, we confirmed that progesterone plays a neuroprotective role after ICH. In particular, the neuroprotective effect of progesterone in ICH may be related to the promotion of axonal regeneration, and the PI3K/AKT/mTOR pathway appears to play a vital role in this process. Therefore, progesterone may have therapeutic potential for treating ICH and other neurodegenerative diseases.

## MATERIALS AND METHODS

### Animals, ICH model, and treatment regimen

In total, 147 male C57BL/6 mice aged 8–10 weeks were provided by the Laboratory Animal Center of Sichuan University. The animals were randomized into the following four groups: sham, ICH, ICH + progesterone, and ICH + progesterone + LY294002. The ICH model was established according to a previous report. The mice were anesthetized with pentobarbital (40 mg/kg intraperitoneally), placed in the prone position, and secured in a stereotaxic frame (RWD Life Science, Shenzhen, China). A small hole for needle insertion was drilled into the exposed skull. Collagenase VII (0.075 units in 0.5 μL saline; Sigma, St. Louis, MO, USA) was injected at the following stereotactic coordinates: 1.0 mm anterior to the bregma, 2.0 mm lateral of the bregma, and 3.5 mm in depth. After injection, the needle was left in position for an additional 10 min.

The mice in the sham group were not administered collagenase or any other reagent. The mice treated with progesterone (Sigma-Aldrich) were given intraperitoneal injections of the calculated dose (8 mg/kg) in 22.5% 2-hydroxypropyl-β-cyclodextrin one hour after surgery and subcutaneously at 6, 24, and 48 hours. In the inhibitor group, LY294002 (10 nmol/2 μL; Selleck Chemicals, Houston, TX, USA) was dissolved in 25% dimethyl-sulfoxide (DMSO) and injected into the lateral ventricle after scalp incision, followed by collagenase 30 min later, and intraperitoneal injections of 8 mg/kg progesterone as described above [16, 20].

### Neurological deficits and MRI

Neurological deficits were evaluated on days one, three, and seven after ICH. The ratings included body symmetry, gait, climbing, circling behavior, front limb symmetry, compulsory circling, and whiskers. Each test was scored on a scale from 0 to 4, with a maximum deficit score of 28. The mice were anesthetized using a 2% isoflurane-air mixture before MRI. A 7.0T MRI scanner (Bruker, Ettlingen, Germany) was used to scan the brain of each group of mice with T2-weighted images, and the main protocol parameters were as follows: repetition time, 100 ms; field of view, 35 × 35 mm; matrix, 256 × 256; echo time, 2.5 ms; slice thickness, 1 mm.

### Brain water content measurement

The intact brains of mice were removed and placed on pre-weighed and numbered tin foil paper. The tissues were weighed on an electronic scale to obtain the wet weight and then dried in an oven at 100° C. The water content of the brain tissue was calculated using the following formula: (wet weight - dry weight) / wet weight × 100%.

### Histological examination

For hematoxylin-eosin staining, the brain sections were deparaffinized and then stained with hematoxylin solution for five minutes. After treatment with hematoxylin differentiation solution, eosin staining was performed for five minutes. The sections were then dehydrated and mounted for observation. For Nissl staining, the sections were deparaffinized and stained with Nissl solution for 20 min. After rinsing with distilled water, differentiation was performed with Nissl Differentiation for 4–8 s. Finally, the sections were dehydrated and mounted for imaging. For TUNEL staining, the operation was performed strictly according to the manufacturer's instructions (Servicebio, Wuhan, China). The fluorescent stain, 4′,6-diamidino-2-phenylindole (DAPI), was used to stain the nuclei, which were then observed under a fluorescence microscope. For LFB staining, the sections were washed with distilled water and stained with 0.1% LFB solution. Then, 0.05% lithium carbonate aqueous solution and 70% alcohol were used for color separation. Finally, the tablets were redyed with 0.25% tar purple solution and several drops of glacial acetic acid, after dehydration.

### Western blotting

The proteins were extracted from the tissue surrounding the hematoma. The concentration was determined by the bicinchoninic acid method, and equal amounts of proteins per sample were denatured and resolved by sodium dodecyl sulfate-polyacrylamide gel electrophoresis (SDS-PAGE). The proteins were transferred to polyvinylidene fluoride (PVDF) membranes, which were blocked with 5% skimmed milk powder or bovine serum albumin for one hour at room temperature, and incubated overnight at 4° C with primary antibodies against NF200 (1:500, CST), GAP43 (1:2000, Servicebio), MAG (1:1000, Servicebio), Nogo-A (1.0 μg/mL, Abcam), Bax (1:4000, Abcam), Bcl-2 (1:2000, Abcam), IL-1β (1:1000, Abcam), IL-6 (1:1000, Abcam), TNF-α (1:1000, Abcam), p-AKT (1:1000, Cell Signaling Technology), AKT (1:1000, Cell Signaling Technology), p-mTOR (1:1000, Abcam), and mTOR (1:10000, Abcam). After the secondary antibody was incubated at room temperature for an hour, enhanced chemiluminescence (ECL) exposure solution was added for observation.

### Immunohistochemistry

The sections were deparaffinized and then boiled in citrate buffer for antigen retrieval. The sections were subsequently processed according to the instructions of the PV9000 immunohistochemistry kit (Servicebio) and incubated overnight at 4° C with anti-GAP43 (1:200, Servicebio) and anti-MAG (1:300, Servicebio) antibodies. After overnight incubation, the sections were washed with phosphate-buffered saline (PBS) and then incubated with secondary antibodies for 30 min at room temperature. Diaminobenzidine (DAB) was added for color development after a PBS wash, and the sections were subsequently counterstained with hematoxylin for 20 s and rinsed under running tap water. After dehydrating and clearing the sections, they were mounted and observed under a microscope. The positively stained regions were analyzed using Image J.

### Immunofluorescence

The slices were incubated with 0.2% Triton for 10 min after rewarming, then blocked with goat serum for an hour and incubated with primary antibodies, including MPO (1:100, Abcam), NeuN (1:100, Abcam), GFAP (1:500, Servicebio), Iba1 (1:1000, Wako), MBP (1:300, Servicebio), and SMI32 (1:200; BioLegend) overnight at 4° C. After washing with PBS, a fluorescent secondary antibody was added dropwise, and the sections were incubated for two hours in the dark. The slides were rewashed, counterstained with DAPI for 10 min, washed with PBS, and mounted with an anti-fluorescence quencher.

### RT-PCR

Total RNA was extracted from the tissue surrounding the hematoma. After reverse transcription, RT-PCR was performed. The primers used were as follows: NF200 forward, GAGTGGTTCCGAGTGAGGTTG; NF200 reverse, GAGTGGTTCCGAGTGAGGTTG; IL-6 forward, CCCCAATTTCCAATGCTCTCC; IL-6 reverse, CGCACTAGGTTTGCCGAGTA; Nogo-A forward, TCAGTGGATGAGACCCTTTTTGC; Nogo-A reverse, GCAGTTTCAAACAGGACAGATGG; glyceraldehyde 3-phosphate dehydrogenase (GAPDH) forward, CCTCGTCCCGTAGACAAAATG; GAPDH reverse, TGAGGTCAATGAAGGGGTCGT.

### Statistical analysis

The statistical analysis was performed using GraphPad Prism software (GraphPad Software, San Diego, CA, USA). All data are presented as the mean ± SEM. Statistical differences between groups were analyzed using unpaired Student's *t*-test, and a P-value < 0.05 was considered statistically significant.

## Supplementary Material

Supplementary Tables
