# Supplementary material for: Progesterone attenuates neurological deficits and exerts a protective effect on damaged axons via the PI3K/AKT/mTOR-dependent pathway in a mouse model of intracerebral hemorrhage
Source: Aging (Albany NY). 2022 Mar 19;14(6):2574–89. doi: 10.18632/aging.203954 (PMC9004566; doi:10.18632/aging.203954)
Supplement: Supplementary Tables [file aging-14-203954-s001.pdf]

## SUPPLEMENTARY TABLES

### Supplementary Table 1. 1, 3, 7 days after ICH was modeled, mice scored in individual task.

| | Body symmetry | Gait | Climbing | Circling behavior | Front limb symmetry | Compulsory circling | Whisker response |
|---|---|---|---|---|---|---|---|
| 1d | 3±0.63 | 2.5±0.55 | 2.67±052 | 2.83±0.75 | 2.83±0.41 | 2.5±0.55 | 2.5±0.55 |
| 3d | 2.17±0.41 | 2±0 | 2±0.63 | 2.5±0.55 | 2.33±0.52 | 2.5±0.55 | 1.83±0.41 |
| 7d | 1.33±0.52 | 1.17±0.41 | 1.17±0.41 | 1.17±0.41 | 1.33±0.52 | 1.17±0.41 | 0.67±0.52 |

### Supplementary Table 2. Mice scored in individual task in ICH and ICH+PROG group.

| | Body symmetry | Gait | Climbing | Circling behavior | Front limb symmetry | Compulsory circling | Whisker response |
|---|---|---|---|---|---|---|---|
| ICH | 1.83±0.41 | 2.33±0.52 | 2.67±0.52 | 2.83±0.41 | 2±0.63 | 2.83±0.41 | 1.33±0.82 |
| ICH+PROG | 1.67±0.75 | 1.83±0.41 | 2.17±0.41 | 1.83±0.41 | 1.33±0.52 | 1.83±0.75 | 1.33±0.52 |
